# Interactive tools for functional annotation of bacterial genomes

**DOI:** 10.1093/database/baae089

**Published:** 2024-09-06

**Authors:** Morgan N Price, Adam P Arkin

**Affiliations:** Environmental Genomics & Systems Biology, Lawrence Berkeley National Laboratory, 1 Cyclotron Rd, Berkeley, CA 94720, United States; Environmental Genomics & Systems Biology, Lawrence Berkeley National Laboratory, 1 Cyclotron Rd, Berkeley, CA 94720, United States

## Abstract

Automated annotations of protein functions are error-prone because of our lack of knowledge of protein functions. For example, it is often impossible to predict the correct substrate for an enzyme or a transporter. Furthermore, much of the knowledge that we do have about the functions of proteins is missing from the underlying databases. We discuss how to use interactive tools to quickly find different kinds of information relevant to a protein’s function. Many of these tools are available via PaperBLAST (http://papers.genomics.lbl.gov). Combining these tools often allows us to infer a protein’s function. Ideally, accurate annotations would allow us to predict a bacterium’s capabilities from its genome sequence, but in practice, this remains challenging. We describe interactive tools that infer potential capabilities from a genome sequence or that search a genome to find proteins that might perform a specific function of interest.

**Database URL**: http://papers.genomics.lbl.gov

## Introduction

For most species of bacteria, we have genome sequences but not much experimental data about their capabilities. So, to understand these bacteria, we look at their genomes and try to predict phenotypes such as how they make energy, which carbon sources they can use, or which amino acids or vitamins they require for growth. In practice, this means predicting the protein-coding genes in the genome and then guessing at those proteins’ functions based on their similarity to proteins of known function (i.e. whose function has been determined experimentally). This commentary describes how to use interactive tools to better predict the functions of proteins from diverse bacteria (and archaea) and the capabilities of those organisms.

**Figure 1.**
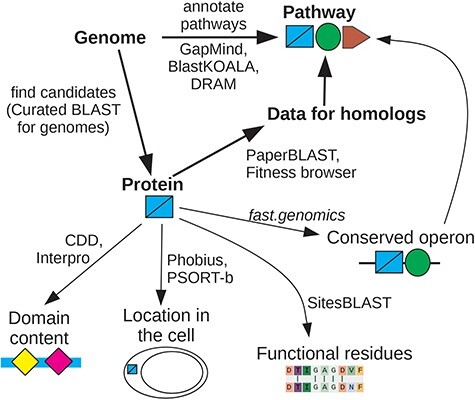
Overview of interactive tools. If you are interested in whether the genome encodes a specific capability, look at pathway annotations from GapMind, or search for candidate proteins that might have a specific function using Curated BLAST for genomes. Once you are interested in a specific protein, search for homologs of known function or for homologs with mutant phenotypes. If data for close homologs are available, this will often suggest a role for the protein. Otherwise, look for protein domains, predicted localization, known functional residues, and conserved operons. These approaches can give complementary hints as to the protein’s function. For details and links, see Supplementary Table S1.

Why are we focusing on interactive tools instead of fully automated annotations? We will show that automated annotations are intrinsically unreliable because much of our knowledge about proteins’ functions is missing from the underlying databases. In practice, automated annotations are often erroneous, even for proteins whose function is known. Automated annotations are necessary because interactive techniques require human attention and cannot be applied to every protein, but we view a protein’s automated annotation as a crude first guess. We focus on interactive tools—ideally, web-based tools that run in a few seconds in a web browser—because these make it easier to consider the wide range of additional data and analyses that can give insight into a protein’s function or a bacterium’s capabilities. An overview of our recommendations is shown in Fig. 1.

This commentary will focus on enzymes and transporters, mostly because we have more experience with annotating metabolism than with other aspects of bacterial physiology. However, these approaches apply to other types of proteins as well. In any case, accurate annotation of enzymes and transporters is central to understanding the metabolic capabilities of diverse bacteria.

## Results

### How accurate are gene annotations for enzymes and transporters?

To estimate the accuracy of gene annotations for enzymes and transporters, we needed a large set of metabolic genes of known function from diverse bacteria. We decided to focus on catabolic genes, as identified using randomly barcoded transposon sequencing (RB-TnSeq [1]). Catabolic genes can usually be identified from RB-TnSeq data because they should be important for fitness during growth with that specific carbon or nitrogen source, but not during growth in most other conditions. Furthermore, a specific molecular function can often be inferred from these mutant phenotypes [2]. For example, if a transporter is specifically important during growth on l-fucose, it is probably an l-fucose transporter. Similarly, if a putative sugar kinase is specifically important during growth on d-glucosamine, then the kinase is probably either a glucosamine kinase or an *N*-acetylglucosamine kinase, depending on whether the organism catabolizes glucosamine via *N*-acetylglucosamine [3]; the correct pathway can usually be identified by examining the other genes that are specifically important for glucosamine utilization.

We selected a random sample of 500 genes with specific phenotypes on carbon or nitrogen sources from the Fitness Browser (http://fit.genomics.lbl.gov [2]). A gene has a specific phenotype in an experiment if the abundance of its mutants changed by at least two-fold, the change is statistically significant, and little change was seen in most other experiments [2]. (These genes are listed in Supplementary Dataset 1.) For each of these 500 genes, we manually determined if the phenotype appeared to be due to a role in the uptake or enzymatic breakdown of that carbon source or nitrogen source. We found 186 enzymes and transporters with specific roles in catabolism whose molecular function could be inferred using the genetic data. The other 314 genes either did not appear to encode enzymes or transporters, or the gene was detrimental to fitness, or the phenotype did not seem so specific, or we could not assign a specific molecular function in catabolism.

Of the proteins with inferred functions in catabolism, about half (96/186) had previously been reannotated in the Fitness Browser [2], but while the Fitness Browser’s reannotations are biased toward novel biology, the 186 proteins represent a random sample of the catabolic functions in the bacteria that we have RB-TnSeq data for. These proteins are from 31 different bacteria. Most of the proteins are from the phylum Pseudomonadota (α,β,γ-proteobacteria), and about half (95/186) are from the genus *Pseudomonas*. These are well-studied taxa, so these proteins should be relatively easy to annotate.

We considered an automated annotation to be correct, or close enough, if it implied the same enzymatic or transport reaction to occur on the substrate that was implied by the mutant phenotype. If the mutant phenotypes implied multiple substrates, and the automated annotation included just one of those substrates, it was still deemed correct. Conversely, if the automated annotation listed additional substrates beyond what was expected from the mutant phenotypes, it was still considered correct: the protein may have other functions beyond those that were captured by the mutant phenotypes.

We considered the lack of a specific annotation to be an error, as a missing annotation could wrongly imply that the organism lacks that activity. In particular, vague annotations such as “sugar kinase” or “multiple sugar transport system” that did not mention any specific substrates were considered as errors, even if the substrate that was inferred from the mutant phenotypes was a sugar. If the tool did not report any annotation, that was also considered an error. Similarly, for fusion proteins with two entirely different activities, the annotation was considered incorrect if it only included one of those activities. (Three of the 186 catabolic proteins are fusion proteins.)

We tested four approaches for automated annotation. First, we used the annotation of the closest homolog from Swiss-Prot, which is a database of ∼570 000 curated annotations [4]. Most of the Swiss-Prot annotations are based on homology, but a significant fraction is supported by experimental evidence. For Swiss-Prot homologs, we required the alignment to cover 70% of both proteins. Second, we used the GhostKOALA tool from Kyoto Encyclopedia of Genes and Genomes (KEGG), which is based on another large database of curated annotations [5]. Third, we used annotations from RefSeq, which are produced by the National Center for Biotechnology Information’s (NCBI’s) Prokaryotic Genome Annotation Pipeline (PGAP [6]). PGAP’s annotations are primarily based on curated descriptions of protein families, as defined by hidden Markov models. Finally, we used CLEAN, which is a machine learning approach based on protein language models and contrastive learning [7]. Since CLEAN is aimed at predicting enzyme function, we ran it on the 114 enzymes only (Supplementary Dataset 1).

If we consider vague or missing annotations to be errors, then annotation errors were common, with accuracies of 50%–69% (Table 1). If we ignore vague or missing annotations, then the accuracy rises to 64%–88%. Enzyme annotations were noticeably more accurate than transporter annotations (79% vs. 54% for Swiss-Prot best hits).

**Table 1.** Accuracy of automated annotations for a random sample of catabolic enzymes and transporters whose function was inferred from mutant phenotypes

		Annotated correctly			
		Swiss-Prot best hit (%)	KEGG (%)	RefSeq (PGAP) (%)	CLEAN (%)
All	186	69	69	50	
Enzymes	114	79	77	61	51
Transporters	72	54	56	33	
Ignore if missing or vague	varies	80	84	88	64
Not Pseudomonadota	13	31	31	15	

Not surprisingly, accuracy was higher when the annotation was transferred from a closer homolog. For Swiss-Prot, if the best homolog was 80% or identical or more, then the annotation was almost always accurate (46/47 proteins). Annotations from homologs that were 50%–80% identical were mostly accurate (85%). However, if the best homolog in Swiss-Prot had under 50% identity, transferring the annotation from the best hit was accurate just 61% of the time. Similarly, enzyme annotations from CLEAN were far more accurate if they were labeled as high confidence (91% vs. 34% otherwise).

**Figure 2.**
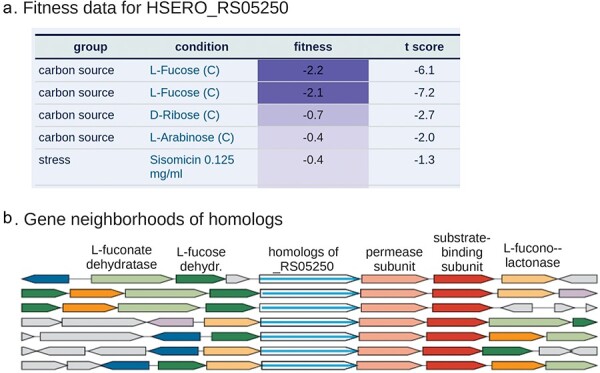
Identification of a l-fucose transporter using RB-TnSeq data and conserved gene neighbors. (a) In *H. seropedicae* SmR1, HSERO_RS05250 is specifically important for l-fucose utilization. This screenshot from the Fitness Browser shows the strongest fitness values for this gene from 96 experiments. A gene’s fitness is the log2 change in the relative abundance of its mutants during an experiment (such as growth in l-fucose). Negative fitness values (dark blue) indicate that mutants are at a disadvantage. (b) Gene neighbors of homologs of HSERO_RS05250 confirm that it is involved in fucose catabolism. This screenshot from “fast.genomics” shows the gene neighborhoods for seven homologous proteins (72%–100% identity) from different genera. Genes are color-coded if similar proteins are present in more than one track. The labels at the top with the likely function of each gene were added by hand. l-fucose dehydrogenase, l-fucono-1,5-lactonase, and l-fuconate dehydratase are the initial steps in an oxidative pathway for fucose catabolism [74]. HSERO_RS05250 is the ATPase subunit of an ABC transporter. The putative permease and substrate-binding subunits are also encoded nearby.

The protein with a different function than expected given its close homolog in Swiss-Prot was HSERO_RS05250, which is a transporter subunit that is specifically important for l-fucose utilization (Fig. 2a). HSERO_RS05250 is 80% identical to A0B297, which Swiss-Prot annotates as acting on ribose, galactose, or methyl galactoside. Using PaperBLAST [8], we could not find any experimental data about the function of close homologs of HSERO_RS05250 or A0B297 besides the RB-TnSeq data. This subfamily is usually encoded near fucose catabolism genes such as l-fucose dehydrogenase, l-fucono-1,5-lactonase, and l-fuconate dehydratase (Fig. 2b). So, we believe that A0B297’s annotation in Swiss-Prot is erroneous. The misannotation of A0B297 is probably based on the experimentally supported functions of the RbsA and MglA proteins of *Escherichia coli*, which are 44%–47% identical to A0B297.

The accuracy of the automated annotations dropped dramatically if we excluded proteins from Pseudomonadota, which is the best-studied phylum of bacteria. The remaining proteins are from Bacteroidota (10 proteins) or Desulfobacterota (3 proteins). Annotation accuracy for these other proteins was just 31%, either for Swiss-Prot best hits or for KEGG. The poor accuracy reflects the lower level of study of those phyla, which leads to a lower similarity of their proteins to characterized proteins. For proteins with homologs in Swiss-Prot, proteins from Pseudomonadota had a median identity to their best hit of 60%, while proteins from other phyla had a median identity of just 39%.

### How up-to-date are automated gene annotations?

The low accuracy of annotation of these 186 proteins is ironic, given that we had previously published the functions for about half of them and that most of the relevant RB-TnSeq data was published >5 years ago [2]. However, as of December 2023, none of these 186 proteins’ functions have been updated based on the RB-TnSeq data, either in Swiss-Prot or in other curated resources of metabolism, such as MetaCyc [9] or BRENDA [10].

The low rate of curation of genes whose function was inferred using RB-TnSeq could reflect skepticism about our approach. To get a broader estimate of the rate at which papers about protein functions are curated, we considered a sample of 32 papers, published in *mBio* in late 2016, that make claims about protein function in their abstract. (This is the subset of the 54 papers analyzed in Price *et al*. [8] that mention the protein’s function in the abstract.) As of January 2024, none of these 32 papers are referred to by curated Swiss-Prot entries. We then asked whether the key proteins were listed as characterized in Swiss-Prot or in other curated databases of experimentally characterized proteins that are incorporated into PaperBLAST (Table 2). We considered not only the original paper but also whether other papers about those proteins’ function had been curated. We found that in 16 cases, the protein(s), or nearly identical orthologs from another strain of the same species, were curated. Overall, in >7 years, half of the characterized proteins have been curated.

**Table 2.** Data sources for the PaperBLAST database

Database	Topic	No. of protein sequences	No. of characterized proteins	References
Text mining	Papers about proteins	697 794	163 615	
Swiss-Prot	All proteins	103 035	102 037	[4]
GeneRIF	Papers about proteins	82 935		[75]
BioLiP	Ligands in structures	38 213		[32]
BRENDA	Enzymes	29 304	29 304	[10]
MetaCyc	Metabolism	12 665	12 664	[9]
ENA /experiment	All proteins	8 775		[69]
CAZy	Carbohydrate metabolism	8 629	8 629	[76]
TCDB	Transporters	8 505	8 505	[70]
CharProtDB	Characterized proteins	7 961	7 173	[77]
EcoCyc	*Escherichia coli*	4 145	3 261	[78]
REBASE	Restriction enzymes	3 818	3 818	[79]
RegPrecise	Transcription factors	3 160		[71]
Fitness Browser reannotations	Mutant phenotypes	1 758	1 757	[2]
PRODORIC	Transcription factors	345	345	[72]

PaperBLAST’s text mining uses EuropePMC [80] to search scientific articles, including the full text of most articles, for protein identifiers. For most of the curated sources (except for EcoCyc and RegPrecise), only proteins with experimental evidence or literature references are included in PaperBLAST [8]. For example, 18% of Swiss-Prot entries have experimental evidence and are included. For TCDB, only transporters with a literature reference and a substrate are included [3]. For the ENA, annotations with the “experiment” tag are further filtered, see the Materials and methods section. To identify characterized proteins, only databases that usually include experimental evidence are included, and heuristics are used to remove uncharacterized proteins [50], such as proteins with functionally uninformative annotations.

The low rate of curation reflects the limited resources available. Swiss-Prot has roughly three full-time biocurators for all prokaryotic proteins, and “tens of thousands of publications remain to be curated for enzymes” [11].

Automated annotations may be even more out of date, relative to current knowledge, because the underlying reference databases may be years out of date. Two of the most popular tools for annotating bacterial genomes are Prokka and the RAST Server: each has >10 000 citations in Google Scholar [12, 13]. As of January 2024, Prokka’s reference database is based on the October 2019 release of Swiss-Prot. The RAST server’s reference databases date back to 2016 or earlier [14].

Finally, in practice, protein annotations are often obtained from RefSeq, GenBank, or TrEMBL (the non-curated part of UniProt). RefSeq updates its annotation pipeline (PGAP) and recomputes annotations every so often, but annotations in GenBank or TrEMBL are rarely updated. Furthermore, annotations in GenBank or TrEMBL have high error rates, probably much higher than those of the automated approaches that we considered [15, 16].

### Why is automated annotation difficult?

Most enzymes and transporters probably belong to known families. For instance, of the 186 catabolic proteins discussed earlier, 172 (92%) have homologs in Swiss-Prot, and even the vague annotations indicate a type of reaction (i.e. “uncharacterized oxidoreductase” or “sugar kinase”) or that the protein is a transporter. Just 10 of the 186 proteins (5%) could not be annotated as the correct type of enzyme, or as a transporter, by using the best hit from Swiss-Prot. However, if a protein from a known family is not closely related to any characterized protein, it is difficult to identify the correct substrate.

To quantify what fraction of bacterial proteins are similar to characterized proteins, we began with a random sample of 2000 protein-coding genes from diverse bacteria. These were taken from representative genomes in “fast.genomics” [17]. We compared these proteins to a database of 189 323 experimentally characterized proteins from all kingdoms of life, which was compiled from 10 different databases in the December 2023 release of PaperBLAST (Table 2). We used ublast [18] to find alignments with an expectation value of ≤10^−10^ and required the alignment to cover 70% of both proteins. We found that just 28% of bacterial proteins are ≥40% identical to a characterized protein. Even at ∼40% identity, annotations for enzymes or transporters will have significant error rates. For instance, for catabolic proteins that were 35%–50% identical to their best hit in Swiss-Prot and for which the best hit had a specific annotation, the error rate was 34%.

Of the bacterial proteins in our sample, most are from genomes of isolates, but 19% are from high-quality metagenome-assembled genomes (MAGs). (“Fast.genomics” uses an MAG as the representative for a genus if it is of high quality and if no high-quality isolate genome is available.) Not surprisingly, proteins from bacterial MAGs are less likely to be ≥40% identical to a characterized protein than proteins from bacterial isolates are (21% vs. 30%, *P* = .0004, Fisher’s exact test).

Archaeal proteins are less likely than bacterial proteins to be similar to a characterized protein. We compared 2000 random protein-coding genes from diverse archaea (again from representative genomes in “fast.genomics”) to our database of characterized proteins. Just 21% of archaeal proteins are ≥40% identical to a characterized protein.

## Discussion

Overall, most prokaryotic proteins do not have a close characterized homolog in the curated databases, which makes it difficult to predict their functions accurately. This underscores the need for interactive tools.

### Finding characterized homologs with PaperBLAST

To determine the likely function of a protein, our first stop is always PaperBLAST, which finds papers about homologs (http://papers.genomics.lbl.gov/ [8]). PaperBLAST takes just a few seconds to compare the query to proteins from 14 curated databases as well as to proteins that are mentioned in scientific articles (Table 2). Ideally, a PaperBLAST search will lead to experimental data about a homolog with a known function, either from one of the curated databases or from a paper that has not been curated yet. As of a few years ago, the odds of finding useful information in PaperBLAST about a vaguely annotated bacterial protein was ∼22% [8]. However, we recommend using PaperBLAST for all proteins, as it can quickly reveal obvious errors in more-specific annotations, such as the l-fucose transporter discussed earlier.

**Figure 3.**
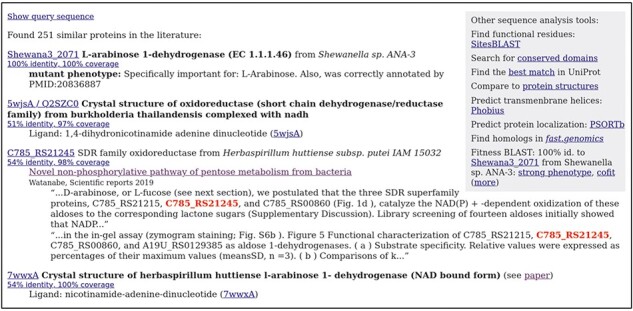
Finding papers about a protein and its homologs with PaperBLAST. This screenshot shows the top results for Shewana3_2071. Experimentally characterized homologs are shown with curated annotations in bold. Homologs that are discussed in papers are shown with snippets of text from those papers. Within each snippet, the homolog’s identifier is highlighted. The top right shows links to other interactive tools.

As another example, consider the dehydrogenase Shewana3_2071 from *Shewanella* sp. ANA-3, which was one of the catabolic proteins in our annotation test. The best hit in Swiss-Prot is a sulfoquinovose 1-dehydrogenase (P0DOV5, 46% identity), but Shewana3_2071 is specifically important for l-arabinose utilization. As shown in Fig. 3, several of the top results from PaperBLAST are informative as to its function. The top result is the reannotation from the Fitness Browser, based on the mutant phenotype. The second result is the crystal structure of a similar protein in the complex with NADH; this confirms that this is a family of dehydrogenases but does not have direct information as to the other substrate. The third result is a paper about C785_RS21245 from *Herbaspirillum huttiense*; the snippet in PaperBLAST’s results mentions functional characterization. That paper shows that the homolog is a 1-aldose dehydrogenase, with a higher specificity constant (*k*_cat_/*K*_M_) for l-arabinose than for other plausible substrates [19]. (The specificity constant for d-fucose was three-fold higher than for l-arabinose, but d-fucose is a rare compound in nature.) The similarity of Shewana3_2071 (54% identity) to a biochemically characterized l-arabinose dehydrogenase (which was published after we made this reannotation) confirms that Shewana3_2071 is an l-arabinose dehydrogenase. On the other hand, the next-best substrate of C785_RS21245, d-xylose, has a specificity constant that is only 14-fold lower than that of l-arabinose. C785_RS21245 itself probably does not contribute significantly to d-xylose degradation: *H. huttiense* has a different d-xylose-specific dehydrogenase whose expression is induced by d-xylose [19]. But in a different genetic context, C785_RS21245 probably could function in d-xylose degradation. This illustrates how enzymes’ annotations often simplify their biochemical capabilities.

To infer a protein’s function from a characterized homolog, the alignment should cover almost the full length of both sequences. If not, examine the domain content (see further). If the proteins are similar over their full length or contain the same domains, then how close is close enough to infer that the protein of interest has the same function as the characterized homolog? As a rule of thumb, for enzymes, the most similar characterized sequence is likely to have the same function if it is >40% identity. (For example, in the annotation test, for enzymes whose best hit in Swiss-Prot was 35%–50% identical and had a specific annotation, the annotation was correct 72% of the time.) It is likely to have a similar function (but perhaps with a somewhat different substrate) if >30% identity. As discussed earlier, transporters are more difficult to annotate, and their specificity evolves more quickly, so a higher %identity is needed to reach the same confidence.

We update the PaperBLAST database every 2 months, so it is much more up-to-date than is possible with automated annotations. Specifically, for each update, we rerun PaperBLAST’s text mining, and we update its copies of Swiss-Prot, GeneRIF, BioLiP, EcoCyc, and the Fitness Browser reannotations. We do not update the other curated databases in PaperBLAST as frequently. Also, PaperBLAST’s copy of MetaCyc is not being updated because it is no longer freely available, and CharProtDB is no longer being updated.

### Viewing mutant phenotypes in the Fitness Browser

PaperBLAST also shows any similarity to proteins with mutant phenotypes from the Fitness Browser. We call this “Fitness BLAST” (see far right of Fig. 3). In particular, PaperBLAST highlights if a homolog has a strong phenotype in a condition (fitness under −2, meaning that mutants decreased in abundance by four-fold or more) or if it has a similar fitness pattern as other genes (“cofitness”).

The Fitness Browser includes RB-TnSeq data from diverse bacteria and archaea [2]. As of February 2024, the Fitness Browser contains 7552 genome-wide experiments from 46 bacteria and two archaea. Most of these bacteria are from the phylum Pseudomonadota, but the Fitness Browser also includes data for four Bacteroidota, two Desulfurbacterota, one Actinomycetota, and one Cyanobacteriota.

We consider a protein to have a functional link, based on its mutant phenotypes, if it has a specific phenotype (as defined earlier, see Fig. 2a for an example) or if it is sufficiently cofit with another gene. Specific phenotypes or cofitness often indicate a functional relationship, especially if they are conserved across similar proteins [2]. To quantify the cofitness of two genes, we use the linear correlation between their fitness profiles. Sufficient cofitness was defined as a correlation of at least 0.8 or a correlation of at least 0.6, and similar proteins in another bacterium have a cofitness of at least 0.6 [2]. Overall, the Fitness Browser contains functional links for 33 261 bacterial proteins. Across the random sample of 2000 proteins from diverse bacteria, 25% have a homolog with a functional link that is >40% identity, with 70% coverage in both ways.

**Figure 4.**
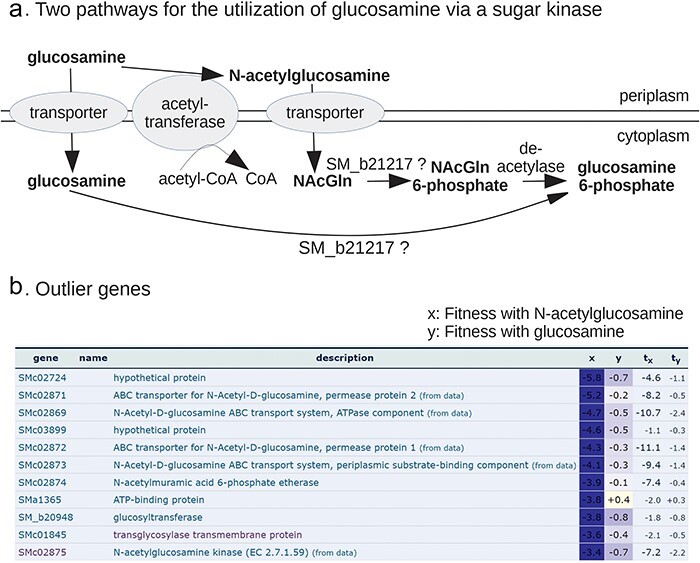
Using the Fitness Browser to consider alternate pathways. (a) Two potential roles for the putative sugar kinase SM_b21217 in glucosamine utilization (adapted from Price *et al*. [3]). (b) A comparison of fitness data from *S. meliloti* with *N*-acetyglucosamine or glucosamine as the nitrogen source. In this screenshot from the Fitness Browser, genes that are important for utilization of *N*-acetyglucosamine (fitness <−2), but not glucosamine, are listed.

To go from a specific phenotype for a putative enzyme to the correct substrate, it is often necessary to consider the rest of the pathway. For example, consider the putative sugar kinase SM_b21217 from *Sinorhizobium meliloti*, which is specifically important for growth with glucosamine as the nitrogen source. Depending on which pathway *S. meliloti* uses to consume glucosamine, SM_b21217 might be a glucosamine kinase or an *N*-acetylglucosamine kinase (Fig. 4a [3]). If *S. meliloti* uses the acetylated pathway, then a transacetylase and a deacetylase would also be involved in glucosamine utilization, and the same transporter and the same kinase might be used during utilization of both glucosamine and *N*-acetylglucosamine (Fig. 4a). The relevant proteins can be found by homology (see Curated BLAST for genomes further) or by using the fitness data. In particular, by examining the genes that are important for utilization of *N*-acetylglucosamine but not glucosamine as the nitrogen source, we can see that *N*-acetylglucosamine kinase and a transporter are important for the utilization of *N*-acetylglucosamine only (Fig. 4b). This suggests that *S. meliloti* does not use the acetylated pathway for glucosamine utilization, and hence that SM_b21217 is a glucosamine kinase. (Another test of which pathway *S. meliloti* uses would be to search for the glucosamine *N*-acetyltransferase using Curated BLAST for genomes, which is described further.)

More broadly, reasoning about the expected phenotypes for a protein will often require examining which other functionally related proteins are present and what their phenotypes are. To support this style of reasoning, the Fitness Browser can render the fitness data in many different ways, including scatterplots for comparing genes’ fitness values, lists of cofit genes, lists of genes with specific phenotypes in a condition, scatterplots for comparing experiments, lists of outlier genes (as in Fig. 4b), heatmaps, and comparisons of the fitness data for similar proteins from different bacteria in the same condition. A related issue that often arises is isozymes. If there are two isozymes for one step in the pathway, then they may be genetically redundant so that neither gene shows the expected phenotype.

One caveat with RB-TnSeq data is the possibility of polar effects: transposon insertions within a gene that is in an operon may disrupt the expression of a downstream gene. If this occurs, then insertions in the gene can have a strong phenotype even though only the downstream gene is important for fitness in that condition. Polar effects are not predominant in the RB-TnSeq data, but they are not rare either [1]. A common sign that a phenotype is due to a polar effect is that the effect depends on which orientation the transposon is inserted in. If the antibiotic resistance marker’s promoter is in the same orientation as the disrupted gene, then the promoter can drive expression of the downstream genes, so polar effects usually occur only for insertions in the opposite orientation. On most pages in the Fitness Browser, clicking on a gene fitness value will show the strain fitness values for insertions in and around the gene, along with the orientation of the antibiotic resistance marker in each strain. Because strain fitness values are much noisier than gene fitness values, the Fitness Browser makes it easy to average the strain fitness value across replicate experiments.

Finally, in our experience, inferring a gene’s likely function from its phenotypes is more straightforward for metabolic genes and transporters than for other types of proteins. Many proteins have pleiotropic phenotypes that are difficult to rationalize, even if the protein is well characterized.

### Checking a protein’s domain content or finding distant homologs

If homologs of known function are not available, or if the alignments do not cover the whole sequence (which suggests that the domain structure may have changed), then we recommend examining the domain structure of the protein of interest, using the Conserved Domain Database (CDD [20]) or InterPro [21]. Searching CDD is fast and can be run interactively. Analyzing a sequence with InterPro is much slower, but precomputed results for all of UniProt (>250 million proteins) are available. For proteins from public bacterial genomes, an identical or nearly identical homolog is probably in UniProt, and these can be found quickly using SANSparallel [22]. Links to CDD and UniProt searches are included in the PaperBLAST results. Either CDD or InterPro will show any similarity of the protein sequence to profiles (hidden Markov models) of protein families, such as from PFam [23]. Profile comparisons are more sensitive than pairwise sequence comparisons, so these tools often find homology that is missed by BLAST-based tools.

Any domains that are present in the characterized homolog but missing from the protein of interest might indicate a loss of function. For example, the characterized protein might have two enzymatic activities, and the protein of interest might have kept just one of them. Conversely, any additional domains in the protein of interest could indicate an additional function. Although domain content is very useful for generating hypotheses about a protein’s function, many domains or families are so broad that they include proteins with very different functions.

Another approach to finding distant homologs is to use the predicted structure and to search for proteins of similar structure. AlphaFold predictions are available for most of the proteins in UniProt [24], and FoldSeek can quickly compare these predictions to other structures and find remote homologs (https://search.foldseek.com/ [25]). Alternatively, if reliable BLAST-level homologs (down to 30% identity or a bit less) are available, then the Jackhammer tool from the HMMer web server can build a profile from the closer homologs and then use that profile to find more distant homologs (https://www.ebi.ac.uk/Tools/hmmer/search/jackhmmer [26]).

If a remote homolog with a known function is found using a tool like FoldSeek, it is interesting to see if the functional residues are conserved. For help finding functional residues, see SitesBLAST further. For aligning distant homologs, we recommend RCSB’s pairwise structure alignment tool (https://www.rcsb.org/alignment [27]).

### Predicting a protein’s location in the cell

Another important aspect of a protein’s function is its location in the cell. In particular, the breakdown of oligosaccharides often begins outside of the cell or in the periplasm. Smaller sugars may also be oxidized or cleaved in the periplasm. Conversely, most other catabolic reactions, and most biosynthetic reactions, take place in the cytoplasm. Understanding where the metabolic enzymes are located is also critical for correctly understanding the role of transporters (Fig. 4a).

To predict a protein’s localization, we recommend using Phobius [28], which predicts signal peptides and transmembrane helices, and PSORTb, which uses a variety of factors including the localization of homologs to predict the localization of prokaryotic proteins [29]. Links to both tools are included in the PaperBLAST results page.

### Finding functional residues with SitesBLAST

Although highly similar sequences are likely to have the same function, an enzyme’s function is not determined by %identity. Rather, most of the protein’s sequence serves to fold the protein into the correct overall shape, and the function is determined by a small number of residues that bind the substrate or participate in catalysis (sometimes called “active site” residues). Unfortunately, most of the popular automated tools for protein annotation do not consider functional residues. (The only exception we are aware of is UniProt’s UniRule [30].)

**Figure 5.**
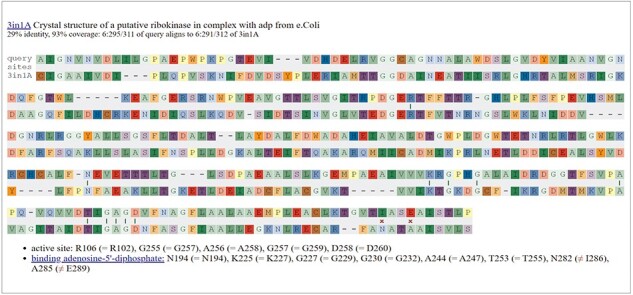
Checking functional residues with SitesBLAST. This screenshot shows the top result for the putative sugar kinase SM_b21217.

Information about these functional residues may be available from SitesBLAST, which makes it easy to see if the functional residues are conserved [31]. For example, the SitesBLAST results for the glucosamine kinase SM_b21217 show that all of the active site residues, and most of the ADP-binding residues, are conserved (Fig. 5). This confirms that SM_b21217 is a kinase.

SitesBLAST takes just a few seconds and compares the input sequence to a database of 170 045 proteins with known functional residues. These functional residues include ligand-binding and active-site residues in crystal structures, as compiled by the BioLiP database [32], and Swiss-Prot features with experimental evidence [4]. SitesBLAST can identify potential functional residues for around half of all proteins [31]. SitesBLAST is available from the PaperBLAST results page or at http://papers.genomics.lbl.gov/sites.

If SitesBLAST shows that the catalytic residues are not exactly conserved, but you still suspect that the protein might be catalytically active, then we recommend checking the Mechanism and Catalytic Site Atlas (https://www.ebi.ac.uk/thornton-srv/m-csa/ [33]). This resource describes the catalytic mechanisms of hundreds of different enzymes, which can help you reason about the potential activity of a protein.

SitesBLAST compares just two proteins. If you want to compare functional residues across many proteins, then we recommend a related tool, Sites on a Tree [31]. Sites on a Tree can indicate whether changes to functional residues are compatible with a conserved function or can identify subfamilies that are likely to have different functions.

### Testing a protein’s function with its structure

If you have a specific hypothesis about the protein’s function, you may be able to use the predicted structure to test your hypothesis. Structural analysis methods are computationally intensive and so are usually not interactive.

Predicted protein complexes can be checked by running AlphaFold on two or three proteins together. We use Google Colab Pro+ to run ColabFold (https://github.com/sokrypton/ColabFold [34]). (No programming is required.) If there are high-confidence contacts between two protein chains, then they probably do form a complex [34].

Docking methods such as AutoDock Vina [35] can in principle be used to predict whether a small molecule binds and where the binding occurs, thereby constraining the substrate specificity of an enzyme or transporter. However, identifying inhibitors or ligands by docking to AlphaFold models is not very accurate [36, 37]. AlphaFold 3 predicts the structure of both the protein and the ligand together, and it predicts the pose (conformation) of the ligand with 75% accuracy [38]. Unfortunately, as of June 2024, the AlphaFold server only supports ∼30 different ions or ligands (http://alphafoldserver.com), and the source code is not available. Still, we hope that the accurate docking for diverse ligands will be available soon. Once poses of potential substrates are identified correctly, it remains challenging to select the correct substrate, as many nonsubstrates will also be predicted to bind [39]. Estimating the energy of binding for the transition state, instead of the substrate, can help to distinguish substrates [39].

### Inferring function from gene neighborhoods or gene presence/absence

Another way to generate hints about a protein’s function is to examine the genes encoded near homologs of the protein of interest. In particular, in bacteria and archaea, genes in conserved operons often have related functions [40–42]. The gene neighborhoods of prokaryotic homologs of a protein of interest can be viewed using “fast.genomics” (http://fast.genomics.lbl.gov/ [17]). A conserved operon will show up as a group of genes that are encoded on the same strand and with a close spacing (usually under 100 nt) across multiple genera (see example in Fig. 2b).

**Figure 6.**
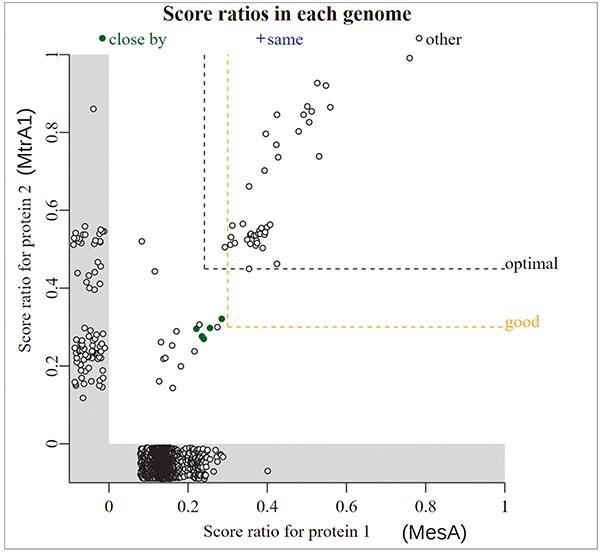
Comparing the presence/absence of two gene families. In this screenshot from “fast.genomics,” each point is a genome. The bit score ratio is the alignment score for the best hit divided by the highest possible alignment score; it is a measure of how similar the best hit (if any) in that genome is to the query. The *x*-axis shows the similarity to MesA from *M. marburgensis*. The *y*-axis shows the similarity to MtrA1 from *M. marburgensis*. Genomes that have one protein, but not the other, are shown in the gray zones below zero. The labels for MesA and MtrA1 were added by hand.

If you have a specific hypothesis about a protein’s function, then which genomes it is found in may also be informative. For instance, an unusual type of methionine synthase was reported in *Methanothermobacter marburgensis* [43]. We will call it MesA [44]. *In vitro*, MesA uses methylcobalamin instead of methyl-tetrahydrofolate as its methyl donor, but with a high *K*_M_ (Michealis–Menten constant), it is expected that a corrinoid (cobalamin-binding) protein would be the physiological donor [43]. We noticed that MesA is found only in methanogens [44], which suggests that MtrA (the corrinoid subunit of tetrahydromethanopterin S-methyltransferase) might be the donor protein. As shown in Fig. 6, genomes that contain reasonably close homologs of MesA, with a bit score of >30% of the maximum, almost always contain MtrA. Also, in a few genomes, the two genes are encoded near each other and on the same strand (filled green points in Fig. 6). This supports a functional relationship between MtrA and MesA. Some genomes with MtrA (score ratio: >0.4) lack MesA; these encode another type of methionine synthase [44].

“Fast.genomics” can go from a protein sequence to homologs from diverse bacteria and archaea in a few seconds. By default, it uses a database of 6377 representative genomes, each from a different genus of bacteria or archaea. Alternatively, it can search within a larger set of genomes (up to 10 representatives per species) within any given taxonomic order. Either way, once it has computed a list of homologs, it can show gene neighborhoods (as in Fig. 2b), compare presence/absence (as in Fig. 6), or show the taxonomic distribution of a gene or a pair of genes.

We believe that “fast.genomics” is usually superior to other fast tools for these comparative genomics analyses because “fast.genomics” compares the query to all genes in the database on the fly (using mmseqs2 [45]). The other fast tools that we are aware of rely on ortholog groups or precomputed groups of similar proteins that ideally have the same function. In practice, ortholog groups are often either too broad—they mix together proteins with different functions—or too narrow so that similar proteins that probably have the same function are missing from the group [17]. Either way, this can lead to less accurate or confusing results. However, “fast.genomics” may not identify remote homologs (roughly, under 30% amino acid identity). For the comparative genomics of broad protein families, such as PFams, we recommend GeCoViz for gene neighborhood analysis (https://gecoviz.cgmlab.org/ [46]) and AnnoTree for co-occurrence analysis [47]. Finally, “fast.genomics” can only compare the presence/absence of two specified proteins. Given a protein family of interest, you can search for other protein families that have a similar occurrence across bacterial genomes using PhyloCorrelate (https://phylocorrelate.uwaterloo.ca/ [48]).

### Finding candidates for a function with curated BLAST for genomes

All the analyses so far began with a protein of interest. However, often we start with a question about a specific organism, such as does it have perchlorate reductase? Can it synthesize leucine or break down phenylacetate? Or maybe we know that it can grow on phenylacetate, and we would like to identify the pathway.

One way to look for a given protein function is to search the automated protein function annotations. However, a protein’s annotation is often nonspecific, even if the protein is similar to a protein that is known to have that function, because of uncertainty as to which of several related functions the protein has.

Instead, Curated BLAST for genomes makes a list of characterized proteins whose annotations match the query, such as “perchlorate” or an Enzyme Commission (EC) number (http://papers.genomics.lbl.gov/curated [49]). (The characterized proteins are taken from PaperBLAST’s database, see Table 2.) Then, it finds all homologs of these proteins in the genome of interest. Candidates can be checked further using PaperBLAST and other tools discussed earlier.

Also, sometimes proteins are missing from the genome annotation, either due to a misprediction of the open reading frame or due to a frameshift error in the genome sequence. After comparing the matching-characterized proteins to the annotated proteins, Curated BLAST for genomes searches the six-frame translation of the genome. This will occasionally find proteins that were not annotated. If there is a frameshift, it is difficult to know if it is a true frameshift that renders the gene nonfunctional (a pseudogene), an error in the sequence, or (less likely) a protein that splits into two pieces that still function. In our experience, frameshift errors are common in genomes that were sequenced solely using long reads (Pacific Biosciences or Oxford Nanopore).

### Tools for annotating pathways

**Figure 7.**
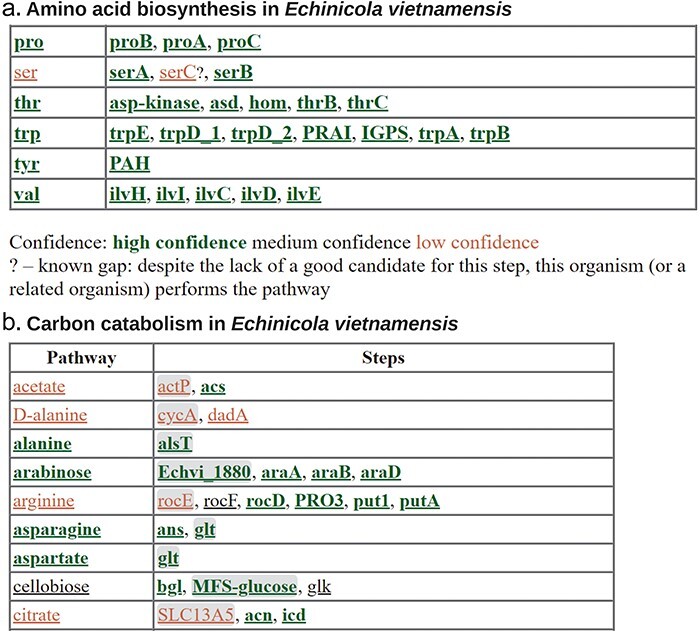
Annotating metabolic pathways with GapMind. (a) Amino biosynthesis in *Echinicola vietnamensis* DSM 17526. This screenshot shows biosynthetic pathways for five of the amino acids along with the key for the color coding. (b) Carbon catabolism in *E. vietnamensis*. This screenshot shows catabolic pathways (or the lack thereof) for nine compounds. Transporters, which are more challenging to annotate, are shown with a gray background.

Searching for individual protein functions can be cumbersome if there are many steps in the pathway of interest, especially if there are multiple alternate pathways. For amino acid biosynthesis and the catabolism of small carbon sources, GapMind annotates the known pathways from bacteria and archaea (http://papers.genomics.lbl.gov/gaps [3, 50]). GapMind does not predict if the capability is present or not; rather, it compares the predicted proteins in the genome to all characterized proteins that carry out relevant enzymatic reactions or transport steps and reports the best-supported path (Fig. 7). Each step is color-coded by whether there is a high-confidence candidate. (Roughly speaking, a high-confidence candidate is at least 40% identical to an experimentally characterized protein that has the function and is less similar to characterized proteins that have other functions.) GapMind is a web-based tool that takes 10–40 s per genome, so it is convenient to run when needed.

There are many other tools with similar goals that cover a different range of pathways than GapMind does. For example, KEGG’s BlastKOALA will annotate enzymes in a genome in ∼15 min (https://www.kegg.jp/blastkoala/ [5]), and the results page includes a pathway-level viewer. Similarly, Distilled and Refined Annotation of Metabolism (DRAM) predicts the presence of metabolic pathways in a prokaryote genome, mostly relating to energy production and central metabolism [51]. DRAM is available within KBase ([52]; http://kbase.us/) and takes ∼15 min.

Regardless of which pathway annotation tool you use, the results are often ambiguous. For instance, what does it mean if the first and third steps in serine synthesis are present, but there is no apparent protein for the second step (Fig. 7a)? In *E. coli*, disrupting any one of the three enzymes results in a requirement for serine for growth, so the missing enzyme is definitely necessary. We refer to these missing steps—which may well be encoded in the genome—as gaps. In some cases, we can infer that the missing step is indeed present: if the other two steps are confidently annotated, and they are not known to be part of a different pathway, then there is no other reason for the two steps to be present. (This logic is stronger if multiple species or genera have the same gap; otherwise, it is possible that the third gene was lost recently and the other two genes, although now useless, have not been lost yet.) Also, GapMind for amino acid biosynthesis identifies “known gaps.” If a related bacterium (from the same family) is known to synthesize the amino acid, even though the gene for the step cannot be found, then the absence of the step should not be viewed as evidence that the pathway is absent. In Fig. 7a, the missing *serC* is a known gap because *E. vietnamensis* grows in a defined minimal medium without any amino acids [50]. *serC* was probably replaced by a non-homologous or distantly related protein that has not been identified yet. More broadly, analyses of gene fitness or gene neighborhoods, as described earlier, can sometimes identify the alternative proteins and fill the gaps [3, 53, 54].

If an organism has a metabolic capability, then at least some of the genes for that capability can usually be identified, but not always. As an extreme example, we have not been able to determine how most Desulfovibrionales make l-serine, despite collecting large-scale genetic data from three representatives [50, 55, 56]. These bacteria do not have a high-confidence assignment for any of the three steps of serine synthesis, yet they grow in a defined minimal medium,

If the above-mentioned tools are not relevant to your question, we recommend using MetaCyc (https://metacyc.org/ [9]) to browse the known metabolic pathways, followed by using Curated BLAST for Genomes to find candidates. Unfortunately, MetaCyc is no longer freely available; if you do not have access, KEGG is an alternative (https://www.genome.jp/kegg/ [57]).

Another way to annotate pathways is to use a genome-scale metabolic model. These models can work well if the model is curated to match the known physiology of the organism, but curation is laborious. Metabolic models that are generated entirely automatically are probably not accurate enough to be useful: in particular, the resulting predictions for amino acid auxotrophies or for carbon source utilization are not at all accurate [58, 59]. In our view, given the challenges of automated annotation discussed earlier, we should expect automatically generated models to be riddled with errors. In principle, automated gap-filling could be used to correct some of these errors, but in practice, automated gap-filling may introduce additional errors [60].

As an alternative to predicting capabilities from the genome, we can also consider the known capabilities of its relatives. For strongly conserved traits, such as many modes of energy production, the taxonomic classification of the organism could be as informative as the genome sequence. For instance, virtually all cyanobacteria can fix carbon dioxide. If a genome of a cyanobacterium appears to be missing a key gene for this pathway, it probably indicates an error in the genome or in the identification of open reading frames. Unfortunately, we do not know of a convenient web-based tool to predict traits from taxonomy; we usually rely on literature searches.

### The future

We expect the volume of high-throughput genetic data to increase dramatically. So far, Fitness BLAST finds reasonably close homologs (≥40% identity) with specific phenotypes or cofitness for about a quarter of all bacterial proteins. We hope to reach the point where useful genetic data are available for homologs of most bacterial and archaeal proteins, but this will require far more data as well as new genetic tools so that randomly barcoded transposon libraries become available for a greater diversity of bacteria and archaea.

We also expect that databases of the metabolic capabilities of bacteria will grow dramatically. Besides enabling more accurate predictions of metabolic capabilities from genome sequences, this will enable the identification of gaps in inferred pathways on a much larger scale. It may also make it easier to fill gaps by comparing the phylogenetic distributions of candidate proteins to those of the gaps.

We hope that the databases of experimentally characterized proteins will improve to cover more of the knowledge that is not currently available to automated tools. Unfortunately, curation is expensive: once a funding agency has spent ∼$100 000 for scientists to characterize a protein, they are not willing to spend ∼$300 for a curator to enter this information into a database [61]. In our view, this is penny-wise and pound-foolish. In any case, as automated text processing improves, curation might become partly automated, and costs should drop. Focusing curator effort on proteins with many homologs, but without close homologs that are characterized according to the curated databases, might also be a way to reduce costs. Alternatively, scientists could curate the functions of proteins when they publish papers about them (discussed in de Crécy-lLagard *et al*. [11]). Also, more papers could be linked to sequences via large-scale analysis of primer sequences, which are most often found in supplementary material [62].

Recently, high-quality structure predictions have become available for most proteins [24]. In principle, these structures could reveal the function of these proteins. So far, we have found structure prediction most useful for testing putative protein complexes and for finding remote homologs. It is not yet clear how to use AlphaFold and related approaches to predict the substrates of enzymes. Assuming that docking improves but accurate substrate prediction remains impossible, one strategy that may become broadly applicable is to combine docking with the gene neighbor method, also known as “pathway docking” [63–65].

## Materials and methods

### Inferring protein functions from specific phenotypes

Five hundred genes with specific phenotypes in carbon sources and nitrogen sources were taken from the December 2023 release of the Fitness Browser and were checked manually. Many of them were quickly discarded because their phenotypes were detrimental, their phenotypes did not show such a specific pattern when checked manually, or neither their annotations (from KEGG or SEED [66]) nor their domain content (from PFam or TIGRFam [67], which are included in the Fitness Browser) suggested that they were enzymes or transporters. Among the remaining proteins, inferred functions for 96 proteins had previously been incorporated into the Fitness Browser’s reannotations; most of these were reported in the supplementary material of Price *et al*. [2, 3]. For another 90 proteins, we inferred functions using the Fitness Browser and the other interactive tools described earlier, especially PaperBLAST.

We double-checked the assigned functions for all proteins that we classified as annotation errors (by any of the four automated methods). This led to two ambiguous cases. First, the putative transporter PfGW456L13_4770 from *P. fluorescens* GW456-L13 is clearly important for l-asparagine utilization, while Swiss-Prot best hit, KEGG, and RefSeq annotate it as a glutamate/aspartate transporter. Since the main asparaginase appears to be cytoplasmic (PfGW456L13_740), l-aspartate transport cannot explain the phenotype of PfGW456L13_4770. However, mutants of PfGW456L13_4770 may also have a subtle defect during glutamate utilization (fitness = −0.9; we usually ignore effects with |fitness| < 1). Since the data suggested that asparagine is the primary substrate, this was still classified as an error for the automated methods. Second, the putative transporter AO356_14110 from *P. fluorescens* FW300-N2C3 is often annotated as “RarD” or as “chloramphenicol-sensitive protein RarD” because it is 48% identical to *E.coli* RarD. The molecular function on RarD is not known. Another member of this family (PA14_19160, 31% identical to AO356_14110) was proposed to be a citrate transporter, as it is genetically redundant with another citrate transporter during growth on citrate [68]. AO356_14110 is specifically important for growth with d-serine as the nitrogen source (fitness = −1.5). Also, a close homolog is specifically important for growth with d-serine as either the carbon source or the nitrogen source (PfGW456L13_1142, 93% identity, fitness = −3.3 to −4.4.) These mutant phenotypes suggest AO356_14110 and PfGW456L13_1142 are involved in d-serine uptake, rather than chloramphenicol resistance. On the other hand, both of these genomes encode dsdX-type d-serine permeases, which are also important for utilizing d-serine. Our preferred explanation is that the rarD-type permeases are partially genetically redundant with dsdX-type permeases, but it is impossible to be sure. In any case, since chloramphenicol resistance seems unlikely to be the main role of these rarD homologs, we still classified those automated annotations as errors.

### Automated annotations

Annotations were derived from Swiss-Prot by using the best hit with *E* ≤ 10^–5^ and 70% coverage (both ways), as identified using NCBI protein BLAST+ against Swiss-Prot release 2023_05 (downloaded on 12 January 2024). KEGG-based annotations were from GhostKOALA run against prokaryotes + eukaryotes + viruses in January 2024. RefSeq annotations were from the corresponding genomes in RefSeq, downloaded in January 2024. Three of the 186 catabolic proteins are from genomes that were not in RefSeq, so we used RefSeq’s annotations of close homologs instead.

When determining the substrates implied by the best hit in Swiss-Prot, we considered the description and also the “Function” field. For instance, the protein PGA1_c29700 is usually annotated as a glycolate dehydrogenase based on its similarity to the *E. coli* GlcF protein. In the fitness data, PGA1_c29700 is important for the utilization of d,l-lactate or d-lactate (but not l-lactate), which suggests that it is a d-lactate dehydrogenase. The Swiss-Prot description for the best hit (which is *E. coli* GlcF) states that GlcF has similar activity with d-lactate as with glycolate, so activity on d-lactate should be expected; thus, the best-hit annotation from Swiss-Prot was considered correct. Similarly, for CLEAN, which reports only the Enzyme Classification (EC) number, we consulted the description of the EC number. In this case, CLEAN assigned the EC number 1.1.99.14, whose description reports that it also acts on d-lactate. Because users of protein annotations usually consider only the textual description, we may have slightly overstated the accuracy of Swiss-Prot best hits and CLEAN. In contrast, for KEGG and RefSeq, we did not look at further information besides the text that was provided by GhostKOALA or by RefSeq itself.

### PaperBLAST and related databases

Since the original publication describing PaperBLAST [8], we have incorporated additional resources into its database for linking proteins to papers. All of the resources are listed in Table 2. The additional resources are the following:

1. BioLiP is a database of biologically relevant ligands in protein structures [32]. All of these ligand-binding proteins are included in PaperBLAST’s database. Even if there is no link to a publication, the fact that the protein bound the ligand can be informative.
2. MetaCyc is a database of metabolism [9]. Only proteins (or complexes) that link to both a sequence and one or more papers are included in PaperBLAST’s database.
3. PaperBLAST incorporates a small subset of the European Nucleotide Archive (ENA [69]) with experimental evidence. PaperBLAST scans nucleotide entries from the “STD” class (roughly, small-scale sequencing projects) for coding sequences (CDS features) with the /experiment tag. The corresponding proteins are included in PaperBLAST’s database if the nucleotide entry links to one or more papers in PubMed. Also, to filter out genes whose transcription or translation was detected, but whose function might not have been studied, entries with >25 such proteins, or papers that link to >25 proteins in this way, are excluded.
4. The incorporation of the experimentally characterized subset of TCDB, the transporter classification database [70], was described previously [3].
5. RegPrecise is a database of predicted regulons, as reconstructed by comparative genomics [71]. Because RegPrecise often includes predictions for several similar transcription factors from related species, we clustered the predicted regulators at 70% identity and 80% coverage with USEARCH [18]. One representative of each cluster is included in PaperBLAST’s database.
6. The Fitness Browser reannotations are a collection of proteins whose function was inferred from RB-TnSeq data (mostly from Price *et al*. [2, 3]).
7. PRODORIC is a database of experimentally characterized regulons [72]. The regulators that have UniProt identifiers were incorporated into PaperBLAST.

The database of characterized proteins (Table 2) is a subset of the full PaperBLAST database. Heuristics for removing uncharacterized proteins were described previously [50].

SitesBLAST uses a separate database, based on BioLiP and Swiss-Prot entries whose sequence features have experimental evidence. To incorporate BioLiP into SitesBLAST, we cluster the protein sequences at 90% identity and 80% coverage with CD-HIT [73]. From each cluster, we select representatives so that every ligand that binds any member of the cluster is included in a structure.

## Supplementary Material

baae089_Supp

## Data Availability

Except for MetaCyc and Google Colab, all of the tools that we discussed are freely available. Up-to-date databases for PaperBLAST, the Fitness Browser, and GapMind are available from their websites. The database for the February 2024 release of the Fitness Browser is archived at figshare (https://doi.org/10.6084/m9.figshare.25236931.v1). (This is identical to the version we used for identifying catabolic genes in bacteria, except that it also includes data from two archaea.) The database for the December 2023 release of PaperBLAST is archived at figshare (https://doi.org/10.6084/m9.figshare.25254562.v1). The 500 proteins with specific phenotypes that we examined and the 186 enzymes and transporters whose functions we identified, as well as the annotations from automated tools, are available in Supplementary Dataset 1 or at http://tinyurl.com/3dzn9f2b. Annotations for the 186 enzymes and transporters are also available in PaperBLAST or from the Fitness Browser.

## References

[1] Wetmore KM , Price MN, Waters RJ et al. Rapid quantification of mutant fitness in diverse bacteria by sequencing randomly bar-coded transposons. *mBio* 2015;6:e00306-15. doi: 10.1128/mBio.00306-15 25968644 PMC4436071

[2] Price MN , Wetmore KM, Waters RJ et al. Mutant phenotypes for thousands of bacterial genes of unknown function. *Nature* 2018;557:503–09. doi: 10.1038/s41586-018-0124-0 29769716

[3] Price MN , Deutschbauer AM, Arkin AP. Filling gaps in bacterial catabolic pathways with computation and high-throughput genetics. *PLoS Genet* 2022;18:e1010156. doi: 10.1371/journal.pgen.1010156 PMC9007349 35417463

[4] Bateman A , Martin M-J, Orchard S, UniProt Consortium. UniProt: the universal protein knowledgebase in 2023. *Nucleic Acids Res* 2023;51:D523–31. doi: 10.1093/nar/gkac1052 36408920 PMC9825514

[5] Kanehisa M , Sato Y, Morishima K. BlastKOALA and GhostKOALA: KEGG tools for functional characterization of genome and metagenome sequences. *J Mol Biol* 2016;428:726–31. doi: 10.1016/j.jmb.2015.11.006 26585406

[6] Haft DH , Badretdin A, Coulouris G et al. RefSeq and the prokaryotic genome annotation pipeline in the age of metagenomes. *Nucleic Acids Res* 2024;52:D762–69. doi: 10.1093/nar/gkad988 37962425 PMC10767926

[7] Yu T , Cui H, Li JC et al. Enzyme function prediction using contrastive learning. *Science* 2023;379:1358–63. doi: 10.1126/science.adf2465 36996195

[8] Price MN , Arkin AP, Langille MGI. PaperBLAST: text mining papers for information about homologs. *mSystems* 2017;2:128. doi: 10.1128/mSystems.00039-17 PMC5557654 28845458

[9] Caspi R , Billington R, Keseler IM et al. The MetaCyc database of metabolic pathways and enzymes—a 2019 update. *Nucleic Acids Res* 2020;48:D445–53. doi: 10.1093/nar/gkz862 31586394 PMC6943030

[10] Chang A , Jeske L, Ulbrich S et al. BRENDA, the ELIXIR core data resource in 2021: new developments and updates. *Nucleic Acids Res* 2021;49:D498–508. doi: 10.1093/nar/gkaa1025 33211880 PMC7779020

[11] de Crécy-Lagard V , Amorin de Hegedus R, Arighi C et al. A roadmap for the functional annotation of protein families: a community perspective. *Database (Oxford)* 2022;2022:baac062. doi: 10.1093/database/baac062 PMC9374478 35961013

[12] Seemann T . Prokka: rapid prokaryotic genome annotation. *Bioinformatics* 2014;30:2068–69. doi: 10.1093/bioinformatics/btu153 24642063

[13] Aziz RK , Bartels D, Best AA et al. The RAST Server: rapid annotations using subsystems technology. *BMC Genomics* 2008;9:75. doi: 10.1186/1471-2164-9-75 PMC2265698 18261238

[14] Davis JJ , Gerdes S, Olsen GJ et al. PATtyFams: protein families for the microbial genomes in the PATRIC database. *Front Microbiol* 2016;7:118. doi: 10.3389/fmicb.2016.00118 PMC4744870 26903996

[15] Schnoes AM , Brown SD, Dodevski I et al. Annotation error in public databases: misannotation of molecular function in enzyme superfamilies. *PLoS Comput Biol* 2009;5:e1000605. doi: 10.1371/journal.pcbi.1000605 PMC2781113 20011109

[16] Rembeza E , Engqvist MKM, Punta M. Experimental and computational investigation of enzyme functional annotations uncovers misannotation in the EC 1.1.3.15 enzyme class. *PLoS Comput Biol* 2021;17:e1009446. doi: 10.1371/journal.pcbi.1009446 PMC8491902 34555022

[17] Price MN , Arkin AP. A fast comparative genome browser for diverse bacteria and archaea. *PLoS One* 2024;19:e0301871. doi: 10.1371/journal.pone.0301871 PMC11003636 38593165

[18] Edgar RC . Search and clustering orders of magnitude faster than BLAST. *Bioinformatics* 2010;26:2460–61. doi: 10.1093/bioinformatics/btq461 20709691

[19] Watanabe S , Fukumori F, Nishiwaki H et al. Novel non-phosphorylative pathway of pentose metabolism from bacteria. *Sci Rep* 2019;9:155. doi: 10.1038/s41598-018-36774-6 PMC6336799 30655589

[20] Marchler-Bauer A , Derbyshire MK, Gonzales NR et al. CDD: NCBI’s conserved domain database. *Nucleic Acids Res* 2015;43:D222–6. doi: 10.1093/nar/gku1221 25414356 PMC4383992

[21] Finn RD , Attwood TK, Babbitt PC et al. InterPro in 2017-beyond protein family and domain annotations. *Nucleic Acids Res* 2017;45:D190–99. doi: 10.1093/nar/gkw1107 27899635 PMC5210578

[22] Somervuo P , Holm L. SANSparallel: interactive homology search against UniProt. *Nucleic Acids Res* 2015;43:W24–9. doi: 10.1093/nar/gkv317 25855811 PMC4489265

[23] Finn RD , Bateman A, Clements J et al. Pfam: the protein families database. *Nucleic Acids Res* 2014;42:D222–30. doi: 10.1093/nar/gkt1223 24288371 PMC3965110

[24] Varadi M , Anyango S, Deshpande M et al. AlphaFold Protein Structure Database: massively expanding the structural coverage of protein-sequence space with high-accuracy models. *Nucleic Acids Res* 2022;50:D439–44. doi: 10.1093/nar/gkab1061 34791371 PMC8728224

[25] van Kempen M , Kim SS, Tumescheit C et al. Fast and accurate protein structure search with Foldseek. *Nat Biotechnol* 2024;42:243–46. doi: 10.1038/s41587-023-01773-0 37156916 PMC10869269

[26] Potter SC , Luciani A, Eddy SR et al. HMMER web server: 2018 update. *Nucleic Acids Res* 2018;46:W200–04. doi: 10.1093/nar/gky448 29905871 PMC6030962

[27] Burley SK , Bhikadiya C, Bi C et al. RCSB Protein Data Bank: powerful new tools for exploring 3D structures of biological macromolecules for basic and applied research and education in fundamental biology, biomedicine, biotechnology, bioengineering and energy sciences. *Nucleic Acids Res* 2021;49:D437–51. doi: 10.1093/nar/gkaa1038 33211854 PMC7779003

[28] Käll L , Krogh A, Sonnhammer ELL. A combined transmembrane topology and signal peptide prediction method. *J Mol Biol* 2004;338:1027–36. doi: 10.1016/j.jmb.2004.03.016 15111065

[29] Yu NY , Wagner JR, Laird MR et al. PSORTb 3.0: improved protein subcellular localization prediction with refined localization subcategories and predictive capabilities for all prokaryotes. *Bioinformatics* 2010;26:1608–15. doi: 10.1093/bioinformatics/btq249 20472543 PMC2887053

[30] MacDougall A , Volynkin V, Saidi R et al. UniRule: a unified rule resource for automatic annotation in the UniProt Knowledgebase. *Bioinformatics* 2021;36:5562. doi: 10.1093/bioinformatics/btaa663 PMC8016456 33821964

[31] Price MN , Arkin AP, Medema M. Interactive analysis of functional residues in protein families. *mSystems* 2022;7:e0070522. doi: 10.1128/msystems.00705-22 PMC9765024 36374048

[32] Zhang C , Zhang X, Freddolino PL et al. BioLiP2: an updated structure database for biologically relevant ligand-protein interactions. *Nucleic Acids Res* 2024;52:D404–12. doi: 10.1093/nar/gkad630 37522378 PMC10767969

[33] Ribeiro AJM , Tyzack JD, Borkakoti N et al. A global analysis of function and conservation of catalytic residues in enzymes. *J Biol Chem* 2020;295:314–24. doi: 10.1074/jbc.REV119.006289 31796628 PMC6956550

[34] Yin R , Feng BY, Varshney A et al. Benchmarking AlphaFold for protein complex modeling reveals accuracy determinants. *Protein Sci* 2022;31:e4379. doi: 10.1002/pro.4379 PMC9278006 35900023

[35] Trott O , Olson AJ. AutoDock Vina: improving the speed and accuracy of docking with a new scoring function, efficient optimization, and multithreading. *J Comput Chem* 2010;31:455–61. doi: 10.1002/jcc.21334 19499576 PMC3041641

[36] Wong F , Krishnan A, Zheng EJ et al. Benchmarking AlphaFold-enabled molecular docking predictions for antibiotic discovery. *Mol Syst Biol* 2022;18:e11081. doi: 10.15252/msb.202211081 PMC9446081 36065847

[37] Lyu J , Kapolka N, Gumpper R et al. AlphaFold2 structures guide prospective ligand discovery. Science 2024;384:eadn6354. doi: 10.1126/science.adn6354 PMC11253030 38753765

[38] Abramson J , Adler J, Dunger J et al. Accurate structure prediction of biomolecular interactions with AlphaFold 3. *Nature* 2024;630:493–500. doi: 10.1038/s41586-024-07487-w 38718835 PMC11168924

[39] Hermann JC , Ghanem E, Li Y et al. Predicting substrates by docking high-energy intermediates to enzyme structures. *J Am Chem Soc* 2006;128:15882–91. doi: 10.1021/ja065860f 17147401

[40] Dandekar T , Snel B, Huynen M et al. Conservation of gene order: a fingerprint of proteins that physically interact. *Trends Biochem Sci* 1998;23:324–28. doi: 10.1016/S0968-0004(98)01274-2 9787636

[41] Huynen M , Snel B, Lathe W et al. Predicting protein function by genomic context: quantitative evaluation and qualitative inferences. *Genome Res* 2000;10:1204–10. doi: 10.1101/gr.10.8.1204 10958638 PMC310926

[42] Wolf YI , Rogozin IB, Kondrashov AS et al. Genome alignment, evolution of prokaryotic genome organization, and prediction of gene function using genomic context. *Genome Res* 2001;11:356–72. doi: 10.1101/gr.161901 11230160

[43] Schröder I , Thauer RK. Methylcobalamin:homocysteine methyltransferase from *Methanobacterium thermoautotrophicum*. Identification as the metE gene product. *Eur J Biochem* 1999;263:789–96. doi: 10.1046/j.1432-1327.1999.00559.x 10469143

[44] Price MN , Deutschbauer AM, Arkin AP. Four families of folate-independent methionine synthases. *PLoS Genet* 2021;17:e1009342. doi: 10.1371/journal.pgen.1009342 PMC7857596 33534785

[45] Mirdita M , Steinegger M, Söding J. MMseqs2 desktop and local web server app for fast, interactive sequence searches. *Bioinformatics* 2019;35:2856–58. doi: 10.1093/bioinformatics/bty1057 30615063 PMC6691333

[46] Botas J , Rodríguez Del Río Á, Giner-Lamia J et al. GeCoViz: genomic context visualisation of prokaryotic genes from a functional and evolutionary perspective. *Nucleic Acids Res* 2022;50:W352–57. doi: 10.1093/nar/gkac367 35639770 PMC9252766

[47] Mendler K , Chen H, Parks DH et al. AnnoTree: visualization and exploration of a functionally annotated microbial tree of life. *Nucleic Acids Res* 2019;47:4442–48. doi: 10.1093/nar/gkz246 31081040 PMC6511854

[48] Tremblay BJ-M , Lobb B, Doxey AC. PhyloCorrelate: inferring bacterial gene-gene functional associations through large-scale phylogenetic profiling. *Bioinformatics* 2021;37:17–22. doi: 10.1093/bioinformatics/btaa1105 33416870

[49] Price MN , Arkin AP, Greene CS. Curated BLAST for genomes. *mSystems* 2019;4:10–128. doi: 10.1128/mSystems.00072-19 PMC6435814 30944879

[50] Price MN , Deutschbauer AM, Arkin AP. GapMind: automated annotation of amino acid biosynthesis. *mSystems* 2020;5:10–128. doi: 10.1128/msystems.00291-20 PMC7311316 32576650

[51] Shaffer M , Borton MA, McGivern BB et al. DRAM for distilling microbial metabolism to automate the curation of microbiome function. *Nucleic Acids Res* 2020;48:8883–900. doi: 10.1093/nar/gkaa621 32766782 PMC7498326

[52] Arkin AP , Cottingham RW, Henry CS et al. KBase: the United States Department of Energy Systems Biology Knowledgebase. *Nat Biotechnol* 2018;36:566–69. doi: 10.1038/nbt.4163 29979655 PMC6870991

[53] Price MN , Zane GM, Kuehl JV et al. Filling gaps in bacterial amino acid biosynthesis pathways with high-throughput genetics. *PLoS Genet* 2018;14:e1007147. doi: 10.1371/journal.pgen.1007147 PMC5764234 29324779

[54] Ashniev GA , Petrov SN, Iablokov SN et al. Genomics-based reconstruction and predictive profiling of amino acid biosynthesis in the human gut microbiome. *Microorganisms* 2022;10:740. doi: 10.3390/microorganisms10040740 PMC9026213 35456791

[55] Trotter VV , Shatsky M, Price MN et al. Large-scale genetic characterization of the model sulfate-reducing bacterium, *Desulfovibrio vulgaris Hildenborough*. *Front Microbiol* 2023;14:1095191. doi: 10.3389/fmicb.2023.1095191 PMC10102598 37065130

[56] Kuehl JV , Price MN, Ray J et al. Functional genomics with a comprehensive library of transposon mutants for the sulfate-reducing bacterium *Desulfovibrio alaskensis* G20. *mBio* 2014;5:e01041–14. doi: 10.1128/mBio.01041-14 24865553 PMC4045070

[57] Kanehisa M , Sato Y, Kawashima M et al. KEGG as a reference resource for gene and protein annotation. *Nucleic Acids Res* 2016;44:D457–62. doi: 10.1093/nar/gkv1070 26476454 PMC4702792

[58] Price M . Erroneous predictions of auxotrophies by CarveMe. *Nat Ecol Evol* 2023;7:194–95. doi: 10.1038/s41559-022-01936-3 36471119

[59] Gralka M , Pollak S, Cordero OX. Genome content predicts the carbon catabolic preferences of heterotrophic bacteria. *Nat Microbiol* 2023;8:1799–808. doi: 10.1038/s41564-023-01458-z 37653010

[60] Karp PD , Weaver D, Latendresse M. How accurate is automated gap filling of metabolic models? *BMC Syst Biol* 2018;12:73. doi: 10.1186/s12918-018-0593-7 PMC6006690 29914471

[61] Karp PD . How much does curation cost? *Database (Oxford)* 2016;2016:baw110. doi: 10.1093/database/baw110 PMC4976296 27504008

[62] Haeussler M , Gerner M, Bergman CM. Annotating genes and genomes with DNA sequences extracted from biomedical articles. *Bioinformatics* 2011;27:980–86. doi: 10.1093/bioinformatics/btr043 21325301 PMC3065681

[63] Zhao S , Kumar R, Sakai A et al. Discovery of new enzymes and metabolic pathways by using structure and genome context. *Nature* 2013;502:698–702. doi: 10.1038/nature12576 24056934 PMC3966649

[64] Calhoun S , Korczynska M, Wichelecki DJ et al. Prediction of enzymatic pathways by integrative pathway mapping. *eLife* 2018;7:e31097. doi: 10.7554/eLife.31097 PMC5788505 29377793

[65] Kumar R , Zhao S, Vetting MW et al. Prediction and biochemical demonstration of a catabolic pathway for the osmoprotectant proline betaine. *mBio* 2014;5:e00933-13. doi: 10.1128/mBio.00933-13 24520058 PMC3950512

[66] Overbeek R , Olson R, Pusch GD et al. The SEED and the Rapid Annotation of microbial genomes using Subsystems Technology (RAST). *Nucleic Acids Res* 2014;42:D206–14. doi: 10.1093/nar/gkt1226 24293654 PMC3965101

[67] Haft DH , Selengut JD, Richter RA et al. Tigrfams and genome properties in 2013. *Nucleic Acids Res* 2013;41:D387–95. doi: 10.1093/nar/gks1234 23197656 PMC3531188

[68] Underhill SAM , Cabeen MT. Redundancy in citrate and *cis*-aconitate transport in *Pseudomonas aeruginosa*. *J Bacteriol* 2022;204:e0028422. doi: 10.1128/jb.00284-22 PMC9765132 36321838

[69] Cummins C , Ahamed A, Aslam R et al. The European Nucleotide Archive in 2021. *Nucleic Acids Res* 2022;50:D106–10. doi: 10.1093/nar/gkab1051 34850158 PMC8728206

[70] Saier MH , Reddy VS, Tsu BV et al. The Transporter Classification Database (TCDB): recent advances. *Nucleic Acids Res* 2016;44:D372–79. doi: 10.1093/nar/gkv1103 26546518 PMC4702804

[71] Novichkov PS , Kazakov AE, Ravcheev DA et al. RegPrecise 3.0—a resource for genome-scale exploration of transcriptional regulation in bacteria. *BMC Genomics* 2013;14:745. doi: 10.1186/1471-2164-14-745 PMC3840689 24175918

[72] Dudek C-A , Jahn D. PRODORIC: state-of-the-art database of prokaryotic gene regulation. *Nucleic Acids Res* 2022;50:D295–302. doi: 10.1093/nar/gkab1110 34850133 PMC8728284

[73] Fu L , Niu B, Zhu Z et al. CD-HIT: accelerated for clustering the next-generation sequencing data. *Bioinformatics* 2012;28:3150–52. doi: 10.1093/bioinformatics/bts565 23060610 PMC3516142

[74] Hobbs ME , Vetting M, Williams HJ et al. Discovery of an L-fucono-1,5-lactonase from cog3618 of the amidohydrolase superfamily. *Biochemistry* 2013;52:239–53. doi: 10.1021/bi3015554 23214453 PMC3542637

[75] Mitchell JA , Aronson AR, Mork JG et al. Gene indexing: characterization and analysis of NLM’s GeneRIFs. *AMIA Annu Symp Proc* 2003;2003:460–64. 14728215 PMC1480312

[76] Lombard V , Golaconda Ramulu H, Drula E et al. The carbohydrate-active enzymes database (CAZy) in 2013. *Nucleic Acids Res* 2014;42:D490–5. doi: 10.1093/nar/gkt1178 24270786 PMC3965031

[77] Madupu R , Richter A, Dodson RJ et al. CharProtDB: a database of experimentally characterized protein annotations. *Nucleic Acids Res* 2012;40:D237–41. doi: 10.1093/nar/gkr1133 22140108 PMC3245046

[78] Karp PD , Paley S, Caspi R et al. The EcoCyc database (2023). *Ecosal Plus* 2023;11:eesp00022023. doi: 10.1128/ecosalplus.esp-0002-2023 PMC10729931 37220074

[79] Roberts RJ , Vincze T, Posfai J et al. REBASE—a database for DNA restriction and modification: enzymes, genes and genomes. *Nucleic Acids Res* 2015;43:D298–9. doi: 10.1093/nar/gku1046 25378308 PMC4383893

[80] Europe PMC Consortium . Europe PMC: a full-text literature database for the life sciences and platform for innovation. *Nucleic Acids Res* 2015;43:D1042–8. doi: 10.1093/nar/gku1061 25378340 PMC4383902

