# Supplementary material for: Interactive tools for functional annotation of bacterial genomes
Source: Database (Oxford). 2024 Sep 6;2024:baae089. doi: 10.1093/database/baae089 (PMC11378808; doi:10.1093/database/baae089)
Supplement: baae089_Supp [file baae089_supp.zip › suppl_data/opinion_TableS1.docx]

| **Name** | **Use** | **Taxonomic range** | **URL** |
| --- | --- | --- | --- |
| PaperBLAST | Find papers about homologs (fast) | any | [papers.genomics.lbl.gov](http://papers.genomics.lbl.gov/) |
| *Linked to by PaperBLAST (fast):* | | | |
| SitesBLAST | Find homologs with known functional sites | any | [papers.genomics.lbl.gov/sites](https://papers.genomics.lbl.gov/sites) |
| Conserved Domain Database (CDD) | Find families that a protein belongs to | any | [https://www.ncbi.nlm.nih.gov/ Structure/cdd/wrpsb.cgi](https://www.ncbi.nlm.nih.gov/Structure/cdd/wrpsb.cgi) |
| Best match in UniProt/InterPro | Find the most similar protein (using kmers and SANSparallel) | any | [https://fast.genomics.lbl.gov/ cgi/bestHitUniprot.cgi](https://fast.genomics.lbl.gov/cgi/bestHitUniprot.cgi) |
| Phobius | Find signal peptides and transmembrane helices | any | [phobius.sbc.su.se](https://phobius.sbc.su.se/) |
| PSORTb | Predict localization in the cell | prokaryotes | [www.psort.org/psortb](https://www.psort.org/psortb/) |
| *fast.genomics* | Browse homologs in diverse prokaryotes | prokaryotes | [fast.genomics.lbl.gov](http://fast.genomics.lbl.gov/) |
| Fitness Browser | Browse RB-TnSeq data | prokaryotes | [fit.genomics.lbl.gov](http://fit.genomics.lbl.gov/) |
| *More tools for individual proteins:* | | | |
| InterProScan | Find families that a protein belongs to (slow) | any | [https://www.ebi.ac.uk/interpro/ search/sequence/](https://www.ebi.ac.uk/interpro/search/sequence/) |
| Jackhmmer | Find distant homologs in representative proteomes (slow) | any | [https://www.ebi.ac.uk/Tools/ hmmer/search/jackhmmer](https://www.ebi.ac.uk/Tools/hmmer/search/jackhmmer) |
| FoldSeek | Find similar structures (fast) | any | [search.foldseek.com](https://search.foldseek.com/) |
| ColabFold | Predict a protein’s structure (slow) | any | [https://github.com/sokrypton/ ColabFold](https://github.com/sokrypton/ColabFold) |
| RCSB pairwise alignment | Align two structures (fast) | any | [www.rcsb.org/alignment](https://www.rcsb.org/alignment) |
| GeCoViz | View conserved gene neighbors (fast) | prokaryotes | [gecoviz.cgmlab.org](https://gecoviz.cgmlab.org/) |
| PhyloCorrelate | Find families with similar occurrences across genomes (fast) | bacteria | [phylocorrelate.uwaterloo.ca](http://phylocorrelate.uwaterloo.ca/) |
| *Tools for genomes:* | | | |
| GapMind | Annotate amino acid biosynthesis or carbon utilization (fast) | prokaryotes | [papers.genomics.lbl.gov/gaps](http://papers.genomics.lbl.gov/gaps) |
| Curated BLAST for Genomes | Find proteins in the genome that might have a given function (fast) | any | [papers.genomics.lbl.gov/ curated](http://papers.genomics.lbl.gov/curated) |
| DRAM | Annotate central metabolism and energy production (slow) | prokaryotes | Available within KBase: [www.kbase.us](http://www.kbase.us/) |
| KEGG’s BlastKOALA | Annotate metabolic pathways (slow) | any | [www.kegg.jp/blastkoala/](http://www.kegg.jp/blastkoala/) |

**Supplementary Table S1: Tools for functional annotation of prokaryotic proteins and genomes.**
